# Final efficacy and safety results and biomarker analysis of a phase 2 study of cabozantinib in Japanese patients with advanced renal cell carcinoma

**DOI:** 10.1007/s10147-022-02283-w

**Published:** 2023-01-03

**Authors:** Noboru Nakaigawa, Yoshihiko Tomita, Satoshi Tamada, Katsunori Tatsugami, Takahiro Osawa, Mototsugu Oya, Hiroomi Kanayama, Yuji Miura, Naoto Sassa, Kazuo Nishimura, Masahiro Nozawa, Naoya Masumori, Yasuhide Miyoshi, Shingo Kuroda, Akiko Kimura

**Affiliations:** 1 Department of Urology, Kanagawa Cancer Center, 2-3-2 Nakao, Asahi-Ku, Yokohama, 241-8515 Japan; 2 Department of Urology, Molecular Oncology, Niigata University Graduate School of Medical and Dental Sciences, 1-757 Asahimachi-Dori, Chuo-Ku, Niigata, 951-8510 Japan; 3 Department of Urology, Bell Land General Hospital, 500-3 Higashiyama, Naka-Ku, Sakai City, Osaka 599-8247 Japan; 4 Department of Urology, Kitakyushu Municipal Medical Center, 2-1-1 Bashaku, Kokurakita-Ku, Kitakyushu, 802-8561 Japan; 5 Department of Renal and Genitourinary Surgery, Hokkaido University, Kita 15, Nishi 7, Kita-Ku, Sapporo, Hokkaido 060-8638 Japan; 6 Department of Urology, Keio University School of Medicine, 35 Shinanomachi, Shinjuku-Ku, Tokyo, 160-8582 Japan; 7 Department of Urology, Tokushima University Graduate School of Biomedical Sciences, 3-18-15 Kuramoto-Cho, Tokushima, 770-8503 Japan; 8 Department of Medical Oncology, Toranomon Hospital, 2-2-2 Toranomon, Minato-Ku, Tokyo, 105-8470 Japan; 9 Department of Urology, Aichi Medical University School of Medicine, Yazako-Karimata 1-1, Nagakute, Aichi 480-1195 Japan; 10 Department of Urology, Osaka International Cancer Institute, 3-1-69 Otemae, Chuo-Ku, Osaka, 541-8567 Japan; 11 Department of Urology, Kindai University Faculty of Medicine, 377-2 Onohigashi, Osakasayama-Shi, Osaka-Fu 589-0014 Japan; 12 Department of Urology, Sapporo Medical University School of Medicine, S1 W16, Chuo-Ku, Sapporo, 060-8543 Japan; 13 Department of Urology and Renal Transplantation, Yokohama City University Medical Center, 3-9 Fukuura, Kanazawa-Ku, Yokohama, 326-0004 Japan; 14 Statistical and Quantitative Sciences, Data Science Institute, Research and Development, Takeda Pharmaceutical Company Limited, 1-1 Doshomachi 4-Chome, Chuo-Ku, Osaka, 540-8645 Japan; 15 Oncology Therapeutic Area Unit for Japan and Asia, Takeda Pharmaceutical Company Limited, 1-1 Doshomachi 4-Chome, Chuo-Ku, Osaka, 540-8645 Japan

**Keywords:** Cabozantinib, Renal cell carcinoma, Tyrosine kinase inhibitor, Biomarker, HGF, Japanese

## Abstract

**Background:**

Cabozantinib was established as the standard of care for the treatment of patients with renal cell carcinoma (RCC) whose disease had progressed after vascular endothelial growth factor receptor tyrosine kinase inhibitor (VEGFR-TKI) therapy in the global randomized trial METEOR. A phase 2 study was conducted to bridge the findings in METEOR to Japanese patients. Here, we report a biomarker analysis and update the efficacy and safety results of cabozantinib treatment.

**Methods:**

Japanese patients with RCC who received at least one prior VEGFR-TKI were enrolled and received cabozantinib 60 mg orally once daily. The primary endpoint was objective response rate. Secondary endpoints included progression-free survival, overall survival, and safety. Exploratory analyses included the relationship between plasma protein hepatocyte growth factor (HGF) levels and treatment responses.

**Results:**

In total, 35 patients were enrolled. The median treatment duration was 58.3 (range 5.1–131.4) weeks. The objective response rate was 25.7% (90% confidence interval [CI] 14.1–40.6). Kaplan–Meier estimate of median progression-free survival was 11.1 months (95% CI 7.4–18.4). The estimated progression-free survival proportion was 73.1% (95% CI 54.6–85.0) at 6 months. Median overall survival was not reached. Adverse events were consistent with those in METEOR and the safety profile was acceptable. Nonresponders to cabozantinib showed relatively higher HGF levels than responders at baseline.

**Conclusions:**

Updated analyses demonstrate the long-term efficacy and safety of cabozantinib in Japanese patients with advanced RCC after at least one VEGFR-TKI therapy. Responders tended to show lower baseline HGF levels ClinicalTrials.gov Identifier: NCT03339219.

**Supplementary Information:**

The online version contains supplementary material available at 10.1007/s10147-022-02283-w.

## Introduction

Renal cell carcinoma (RCC) is a heterogenous malignancy and the survival of patients with advanced and/or metastatic RCC is very poor [1, 2]. Understanding the biological basis of RCC has led to the development of new targeted agents, including immune checkpoint inhibitors, vascular endothelial growth factor receptor (VEGFR) inhibitors, tyrosine kinase inhibitors (TKIs), and mammalian target of rapamycin (mTOR) inhibitors [3–8]. Clear cell (cc) RCC commonly involves mutations in the tumor suppressor Von Hippel–Lindau gene, triggering a decrease in the degradation of hypoxia-inducible factor and an increase in VEGF transcription, which leads to tumor angiogenesis [9]. Thus, inhibitors that target VEGFR, which include sorafenib, sunitinib, pazopanib, axitinib, and lenvatinib, are thought to be effective options with ccRCC patients and in recent years, their combination with IO agents has been established as the standard of care for first-line treatment [10]. However, resistance to VEGF-targeted therapies often arises owing to the upregulation of alternative pro-angiogenic and pro-invasive signaling pathways, including the MET and AXL pathways [11, 12]. Therefore, treatment following prior VEGFR-TKI therapy remains a challenge.

Cabozantinib is a receptor TKI with targets that include MET (c-MET), VEGFR2, RET, AXL, KIT, and TIE-2, which are implicated in tumor growth, metastasis, and angiogenesis [13]. In the phase 3, randomized METEOR trial (ClinicalTrials.gov Identifier: NCT01865747), treatment with cabozantinib improved progression-free survival (PFS), overall survival (OS), and objective response rate (ORR) were compared with treatment with everolimus in patients with metastatic RCC who were treated with at least one prior VEGFR-TKI [14]. Plasma samples from 621 to 658 randomized patients were examined for CA9, hepatocyte growth factor (HGF), MET, growth arrest-specific 6 (GAS6) protein, AXL, VEGF, VEGFR2, and interleukin-8 (IL-8) with the aim of identifying potential prognostic or predictive biomarkers. Most of those candidates were biologically relevant to RCC and included cabozantinib receptor targets and their ligands. In a univariate analysis, low baseline HGF, AXL, and VEGF were prognostic markers for improvements in PFS and OS with cabozantinib [15]. Conversely, a multivariable analysis including International Metastatic Renal Cell Carcinoma Database Consortium (IMDC) risk groups (favorable, intermediate, or poor) found that low baseline HGF, GAS6, and VEGF were independently prognostic for improved OS with cabozantinib. As a consistent result, low baseline HGF, a key ligand of MET, correlated with improved PFS and OS with cabozantinib treatment. Regardless of the baseline levels of the biomarkers tested, PFS and OS were favorable with cabozantinib versus everolimus [15].

This phase 2 trial (ClinicalTrials.gov Identifier: NCT03339219) was designed to evaluate the efficacy and safety of cabozantinib in Japanese patients with RCC who met similar criteria to those who participated in the METEOR trial [16]. The study met its primary endpoint after short-term follow-up (data cut-off 23 October 2018: ORR, 20.0% (90% confidence interval [CI] 9.8–34.3); clinical benefit rate (CBR), 85.7% (95% CI 69.7–95.2). Cabozantinib has been approved in Japan mainly based on the results from the METEOR study and the Japanese Phase 2 study.

Here, we update our previous report [16] based on the data collected after the initial cut-off date through to the final database lock date (November 19, 2020). Additionally, exploratory analyses included the relationship between plasma levels of HGF, MET, GAS6, and AXL (examined in the METEOR trial) at baseline and treatment responses.

## Materials and methods

### Study design

This phase 2, open-label, multicenter (19 sites), single-arm trial was conducted to assess the efficacy and safety of cabozantinib in Japanese patients with ccRCC who had received at least one prior VEGFR-TKI therapy [16]. The study protocol and associated documentation were reviewed by institutional review boards at each site. The study was carried out in compliance with the Declaration of Helsinki, the International Council for Harmonisation Good Clinical Practice guidelines, and all applicable local regulations. All patients provided written informed consent before enrollment.

### Patients

Patients who met the following inclusion criteria were enrolled in the study: (1) aged ≥ 20 years with a documented histological or cytological diagnosis of ccRCC; (2) measurable disease as per Response Evaluation Criteria in Solid Tumors version 1.1 (RECIST 1.1) and determined by investigators; (3) had received at least one prior VEGFR-TKI therapy (e.g., sorafenib, sunitinib, axitinib, pazopanib, and tivozanib). Further detailed inclusion and exclusion criteria are described in our previous report [16].

### Study treatment

Patients received cabozantinib 60 mg once daily orally in the fasted state. Treatment was continued if patients showed clinical benefits or until there were any reasons for treatment discontinuation, including death, adverse events (AEs), clinical deterioration, or a second determination of progressive disease (PD). Dose modifications were permitted for managing AEs with two reduced dose levels (40 mg and 20 mg).

### Assessment

Patients were screened within 28 days before the first day of study drug administration (week 1 day 1 [W1D1]). Throughout the study, patient conditions were assessed by the physician and evaluated for efficacy, safety, and health-related quality of life (HRQoL) at scheduled visits. Computed tomography or magnetic resonance imaging of the chest/abdomen/pelvis were performed at screening, 8-week intervals (± 7 days) after W1D1 until the end of the 12-month period, and at 12-week intervals after completion of the first 12 months. Blood and urine samples were obtained every 4 weeks after W1D1.

Questionnaires were collected every 4 weeks after W1D1 until the end of the 9-week period. After week 9, questionnaires were collected every 4 weeks (± 7 days) up to 6 months, and every 8 weeks (± 7 days) until the last tumor assessment.

### Endpoints

The primary endpoint was independent review committee (IRC)-assessed ORR, defined as the proportion of patients with complete response (CR) or partial response (PR) as per RECIST 1.1 with a similar observation at least 28 days later. Secondary endpoints included CBR, OS, PFS, and safety evaluation. OS was determined to be the time to death due to any cause occurring after the first dose and PFS was defined as the time from starting treatment to PD as per RECIST 1.1 or death. Safety was assessed based on symptoms and clinical laboratory parameters at a central laboratory. AE severity was graded by the investigators according to Common Terminology Criteria for Adverse Events (CTCAE; Version 4.03). Treatment emergent AEs (TEAEs) were defined as AEs occurring from the first dose to 30 days after receiving the last dose of the study drug.

Other endpoints included HRQoL assessment by the Functional Assessment of Cancer Therapy-Kidney Cancer Symptom Index 19 Item Version (FKSI-19). An exploratory examination of the protein levels of HGF, MET, GAS6, and AXL was performed.

### Detection of potential biomarkers

Plasma samples collected on W1D1, week 5 day 1 (W5D1) and week 9 day 1 (W9D1) were tested for potential biomarkers, including HGF, MET, GAS6, and AXL (the protein level at W1D1 was defined as baseline). Detection was achieved using commercially available enzyme-linked immunosorbent assay (ELISA) kits and the following procedures: GAS6 (Human Gas 6 DuoSet ELISA, R&D Systems, Inc., Minneapolis, USA), AXL (Human Axl DuoSet ELISA, R&D Systems, Inc., Minneapolis, USA), HGF (Human HGF Immunoassay, R&D Systems, Inc., Minneapolis, USA), and c-MET (Human c-Met Assay Kit MCM, Immuno-Biological Laboratories Co., Ltd., Gunma, Japan). ELISA kits were validated by using test samples over the range of the standard curve, testing for precision and accuracy, examining diurnally, assessing between-day reproducibility and dilution reproducibility, undertaking freeze–thaw tests and stability tests at room temperature, as well as assessing storage stability for up to 6 months. Validation and measurements were conducted by LSI Medicine Corporation (Tokyo, Japan). Plasma protein concentrations (ng/mL) were obtained by fitting to the standard curve.

### Statistical methods

The study aimed to enroll approximately 35 patients, assuming a 10% dropout rate, to provide 32 patients for the analysis of IRC-assessed ORR. The full analysis set (FAS) was used for efficacy analyses, including all patients having received at least one dose of cabozantinib. The safety analysis set used for safety analyses was the same as the FAS. ORR was assessed as point estimates and two-sided 90% exact Clopper–Pearson CIs. The PFS and OS were estimated by Kaplan–Meier analysis. Safety was descriptively summarized. HRQoL values and changes from baseline were descriptively summarized. As an exploratory post hoc analysis, changes from baseline in plasma biomarker levels were evaluated over time at each time point. The differences in baseline plasma biomarker levels between responders and nonresponders were evaluated by two-sample *t* tests. For descriptive purposes, *p* < 0.05 was considered statistically significant for each biomarker test result.

## Results

### Patient disposition

A total of 35 patients with a median age of 63.0 (range 42–84) years were enrolled and all these patients were included in the FAS and the safety analysis set. Of the 35 patients, 15 patients (42.9%) had received immune-oncology (IO) agents. Twenty-four (68.6%) patients had received one prior VEGFR-TKI and eight (22.9%) patients had received two. The most common prior VEGFR-TKI therapies in the total patient group were sunitinib (68.6%) and axitinib (51.4%).

Baseline IMDC risk scores were favorable for 6 (17.1%) patients, intermediate for 22 (62.9%) patients, and poor for 7 (20%) patients [16]. Further detailed baseline characteristics are described in our previous report [16].

### Study drug exposure

**Fig. 1:**
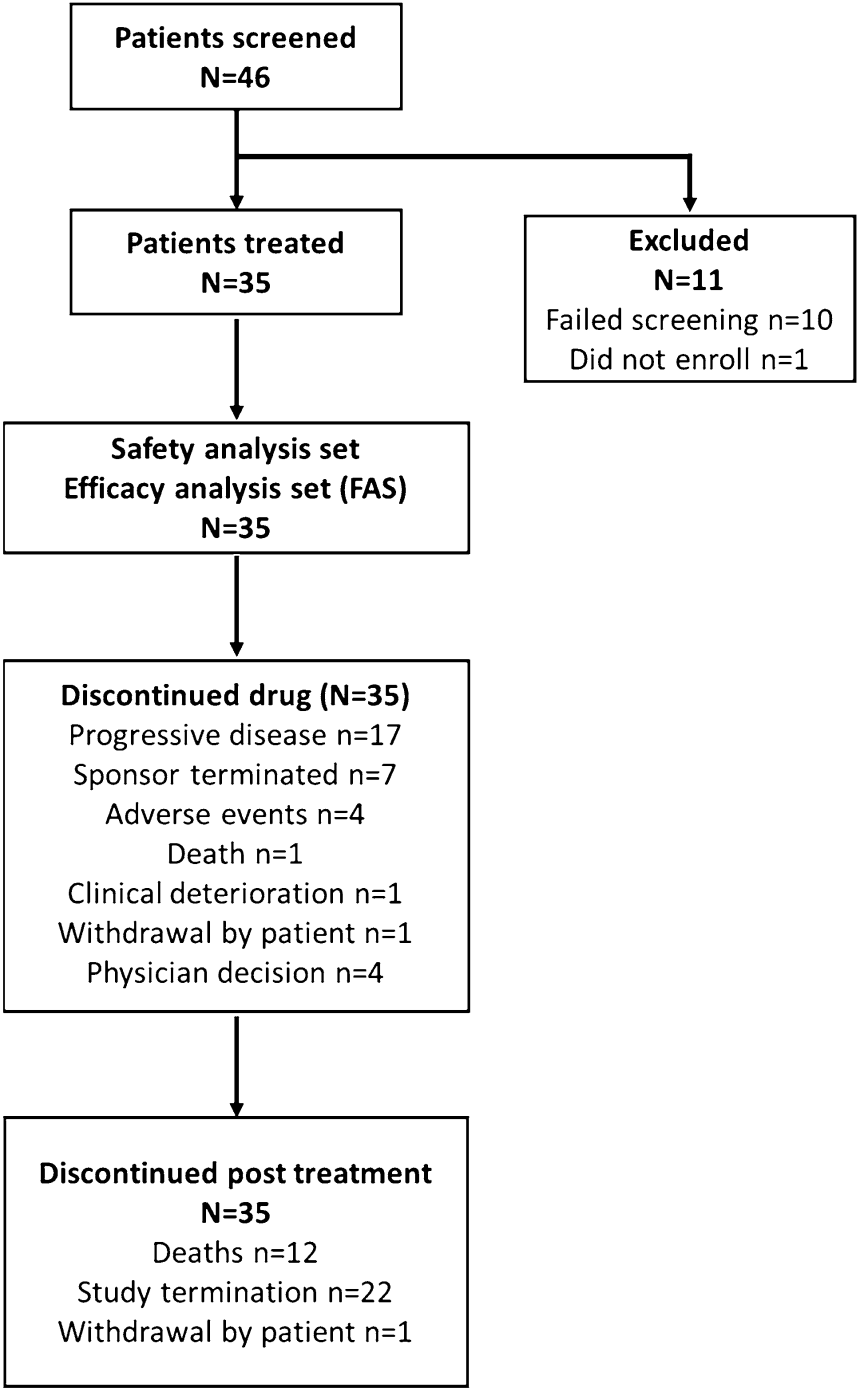
Profile of study enrollment

The median duration of exposure to the study drug was 58.3 weeks (range 5.1–131.4). The median average daily dose was 23.2 mg (range 9.9–60.0 mg) and the corresponding median relative dose intensity was 38.7% (range 16.5–100.0%). All 35 patients discontinued the trial at the point of data cut-off, which included death (*n* = 1), PD (*n* = 17), AEs (*n* = 4), and sponsor termination due to marketing approval of cabozantinib (*n* = 7) (Fig. 1).

### Efficacy assessment

IRC-assessed responses were observed in 9 (25.7%) patients (ORR 25.7%; 90% CI 14.1–40.6), in whom all objective responses were PRs (Table 1). Four (11.4%) patients and 21 (60.0%) patients had progressive disease (PD) and stable disease (SD) as best overall response (BOR), respectively.

**Table 1:** Tumor response, as per RECIST 1.1 by IRC (FAS)

Best overall response^a^	Overall, *N* = 35
CR	0
PR	9 (25.7)
SD	21 (60.0)
PD	4 (11.4)
Not evaluable	0
Missing	1 (2.9)
ORR	
*n* (%)	9 (25.7)
90% CI	14.1, 40.6

Two subjects whose best overall response was non-CR/non-PD were counted as SD in the summary table

*CI* confidence interval, *CR* complete response, *FAS* full analysis set, *IRC* independent review committee, *ORR* objective response rate, *PD* progressive disease, *PR* partial response, *RECIST 1.1* Response Evaluation Criteria in Solid Tumors version 1.1, *SD* stable disease

^a^Data are *n* (%) unless otherwise stated

**Fig. 2:**
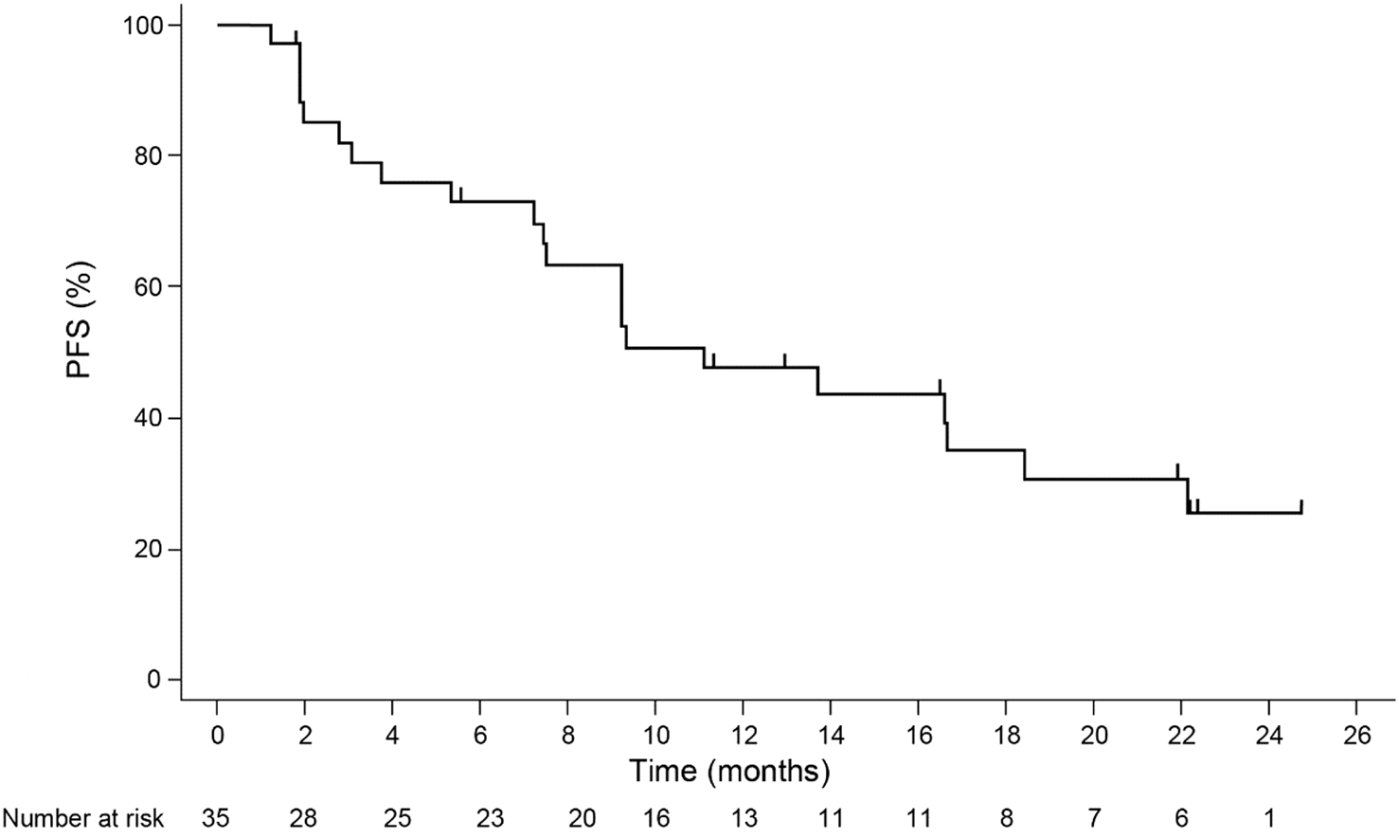
Kaplan–Meier plot of PFS as per RECIST 1.1 by IRC (FAS). *FAS* full analysis set, *IRC* independent review committee, *PFS* progression-free survival, *RECIST 1.1* Response Evaluation Criteria in Solid Tumors version 1.1

Figure 2 shows a Kaplan–Meier plot of PFS by IRC. A total of 22 PFS events occurred. The follow-up time from enrollment of the last patient through to the last procedure for collection of tumor assessment data was approximately 20 months. The median PFS was 11.1 months (95% CI 7.4–18.4). The proportion of PFS was 73.1% (95% CI 54.6–85.0) at 6 months. The investigator-assessed PFS was similar to that by IRC.

**Fig. 3:**
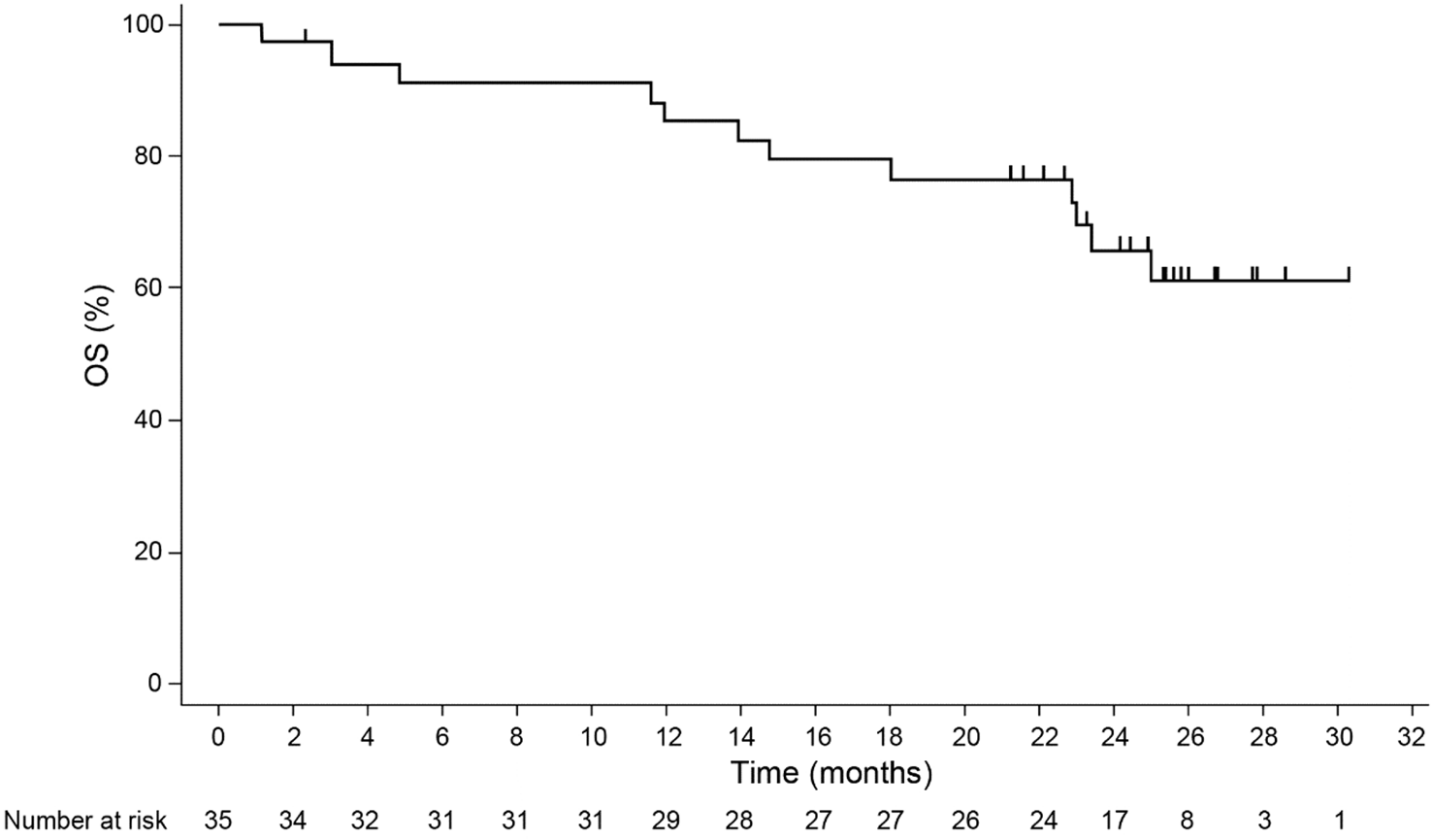
Kaplan–Meier plot of OS (FAS). *FAS* full analysis set*, OS* overall survival

A Kaplan–Meier plot of OS is shown in Fig. 3. A total of 12 deaths occurred during the study and the median OS was not reached. The 1-year and 2-year overall survival rates were 85.4% (95% CI 68.35–93.64%) and 65.7% (95% CI 46.34–79.52%), respectively. The follow-up time from enrollment of the last patient through to the last contact of the last patient was approximately 26 months.

### Safety assessment

TEAEs occurred in ≥ 10% of patients are shown in Table 2. All patients experienced at least one AE and a total of 29 patients reported TEAEs of ≥ Grade 3 severity. The dose of study drug was modified (reduced or interrupted) in 33 patients (94.3%) owing to AEs. The most common AEs reported by ≥ 30% of patients were diarrhea, palmar-plantar erythrodysesthesia syndrome, proteinuria, dysgeusia, hypertension, stomatitis, weight decreased, hepatic function abnormal, decreased appetite, and malaise. Most of those symptoms were assessed as drug-related TEAEs. A total of 15 (42.9%) patients experienced one or more serious adverse events (SAEs) and six of whom experienced drug-related SAEs: cholecystitis (*n* = 2), pneumonia (*n* = 1), pancreatic enzymes increased (*n* = 1), hypocalcemia (*n* = 1), and pneumothorax (*n* = 1). Study treatment was permanently discontinued in 4 (11.4%) patients owing to AEs that were not related to the disease in the study: proteinuria (*n* = 2), gastric fistula (*n* = 1), and pneumothorax (*n* = 1).

**Table 2:** Summary of TEAEs experienced by ≥ 10% of patients

	Cabozantinib-2001 Overall *N* = 35	
Study start day^a^	December 13, 2017	
Data cut off day	November 19, 2020	
Preferred term/grade	All	≥ Grade 3
Patients with any TEAEs, *n* (%)	35 (100.0)	29 (82.9)
Diarrhea	24 (68.6)	3 (8.6)
Palmar-plantar erythrodysesthesia syndrome	23 (65.7)	3 (8.6)
Proteinuria	17 (48.6)	4 (11.4)
Dysgeusia	15 (42.9)	0
Hypertension	15 (42.9)	5 (14.3)
Stomatitis	15 (42.9)	1 (2.9)
Weight decreased	14 (40.0)	1 (2.9)
Hepatic function abnormal	13 (37.1)	3 (8.6)
Decreased appetite	11 (31.4)	3 (8.6)
Malaise	11 (31.4)	0
Back pain	10 (28.6)	0
Constipation	10 (28.6)	0
AST increased	9 (25.7)	1 (2.9)
Nasopharyngitis	8 (22.9)	0
Pyrexia	8 (22.9)	1 (2.9)
ALT increased	7 (20.0)	1 (2.9)
Cancer pain	7 (20.0)	1 (2.9)
Nausea	7 (20.0)	0
Rash	7 (20.0)	0
Anaemia	6 (17.1)	2 (5.7)
Dysphonia	6 (17.1)	0
Fatigue	6 (17.1)	3 (8.6)
Hair colour changes	6 (17.1)	0
Hypothyroidism	6 (17.1)	0
Vomiting	6 (17.1)	0
Amylase increased	5 (14.3)	1 (2.9)
Blood thyroid stimulating hormone increased	5 (14.3)	0
Epistaxis	5 (14.3)	0
Insomnia	5 (14.3)	0
Blood ALP increased	4 (11.4)	0
Cough	4 (11.4)	0
Dental caries	4 (11.4)	0
Dyspnoea	4 (11.4)	0
Gingivitis	4 (11.4)	1 (2.9)
Muscle spasms	4 (11.4)	0
Periodontal disease	4 (11.4)	0

	METEOR Cabozantinib arm *N* = 331		
Study start day^a^	August 08, 2013		
Data cut off day	May 22, 2015		
Preferred term/grade	Grade 1–2	Grade 3	Grade 4
Patients with any TEAEs, *n* (%)	70 (21)	210 (63)	25 (8)
Diarrhea	206 (62)	43 (13)	0
Fatigue	159 (48)	36 (11)	0
Nausea	158 (48)	15 (5)	0
Decreased appetite	146 (44)	10 (3)	0
Palmar-plantar erythrodysesthesia syndrome	115 (35)	27 (8)	0
Vomiting	106 (32)	7 (2)	0
Weight decreased	105 (32)	9 (3)	0
Constipation	89 (27)	1 (< 1)	0
Dysgeusia	80 (24)	0	0
Hypothyroidism	76 (23)	0	0
Hypertension	73 (22)	49 (15)	0
Dysphonia	68 (21)	2 (1)	0
Cough	67 (20)	1 (< 1)	0
Stomatitis	65 (20)	8 (2)	0
Mucosal inflammation	60 (18)	5 (2)	0
Dyspnoea	56 (17)	10 (3)	0
AST increased	55 (17)	5 (2)	0
Back pain	54 (16)	8 (2)	0
Rash	52 (16)	2 (1)	0
Asthenia	49 (15)	15 (5)	0
Abdominal pain	48 (15)	12 (4)	0
ALT increased	47 (14)	7 (2)	1 (< 1)
Pain in extremity	46 (14)	5 (2)	0
Muscle spasms	45 (14)	0	0
Arthralgia	43 (13)	1 (< 1)	0
Headache	43 (13)	1 (< 1)	0
Anaemia	42 (13)	19 (6)	0
Dizziness	41 (12)	1 (< 1)	0
Dyspepsia	40 (12)	1 (< 1)	0
Oedema peripheral	39 (12)	0	0
Hypomagnesaemia	38 (12)	6 (2)	10 (3)
Dry skin	37 (11)	0	0
Proteinuria	37 (11)	8 (2)	0
Flatulence	33 (10)	0	0
Insomnia	32 (10)	0	0

*TEAE* treatment emergent adverse event, *ALP* alkaline phosphatase, *ALT* alanine aminotransferase, *AST* aspartate aminotransferase

ªStudy start day; the actual date on which the first participant was enrolled in a clinical study

A total of 12 deaths were reported in the safety analysis set. Of these, one death occurred within 30 days after the last dose of the study drug; the death was attributed to disease progression. The other 11 deaths occurred beyond 30 days after the last dose of the study drug and were all attributed to disease progression.

Clinically abnormal serum chemistry parameters detected during treatment are summarized in Table 3.

**Table 3:** Clinically significant abnormal serum chemistry laboratory values during treatment by CTCAE Grade (safety analysis set)

Preferred term	Overall *N* = 35, *n* (%)		
	Grade 1–4	Grade 3	Grade 4
ALT increased	26 (74.3)	3 (8.6)	0
AST increased	28 (80.0)	4 (11.4)	0
ALP increased	23 (65.7)	3 (8.6)	0
Albumin decreased	31 (88.6)	1 (2.9)	0
Amylase increased	20 (57.1)	3 (8.6)	0
Corrected calcium increased	2 (5.7)	0	2 (5.7)
Corrected calcium decreased	3 (8.6)	2 (5.7)	0
Creatinine increased	34 (97.1)	0	0
GGT increased	19 (54.3)	2 (5.7)	0
Glucose increased	22 (62.9)	1 (2.9)	0
Glucose decreased	2 (5.7)	0	0
LDH increased	35 (100)	3 (8.6)	0
Lipase increased	16 (45.7)	3 (8.6)	3 (8.6)
Magnesium increased	5 (14.3)	1 (2.9)	0
Magnesium decreased	27 (77.1)	2 (5.7)	1 (2.9)
Phosphate decreased	19 (54.3)	5 (14.3)	0
Potassium increased	5 (14.3)	2 (5.7)	0
Potassium decreased	8 (22.9)	0	0
Sodium increased	0	0	0
Sodium decreased	17 (48.6)	0	0
Total bilirubin increased	6 (17.1)	0	0

*ALP* alkaline phosphatase, *ALT* alanine aminotransferase, *AST* aspartate aminotransferase, *CTCAE* common terminology criteria for adverse events, *GGT* gamma-glutamyl transferase, *LDH* lactate dehydrogenase

### HRQoL assessment results

Mean changes from baseline in FKSI-19 total scores are shown in Supplementary Fig. 1. The mean FKSI-19 total score at baseline was 59.3 (± 10.17). Over the course of treatment, the mean change from the baseline ranged from − 7.0 to − 0.3. Small increases were observed in the scores of emotional status and functional/well-being status over time. The mean score for treatment side effects decreased at all time points. The mean score for disease-related symptoms decreased at almost all time points (Supplementary Table 1).

### Plasma levels of potential biomarkers

Of the 35 patients in the study, post-baseline radiographic tumor assessment was not available for 1 patient and assessment of non-CR/non-PD was made in 2 patients. Additionally, 1 patient with SD with tumor shrinkage was missing baseline biomarker measurements and was omitted from the analysis.

The fold changes from baseline in plasma levels of HGF, MET, GAS6, and AXL at week 5 and week 9 are shown in Table 4. The fold changes of each biomarker (mean) were HGF (1.04, 1.02), MET (1.14, 1.17), GAS6 (1.47, 1.32) and AXL (1.29, 1.35) from baseline to W5D1 or W9D1. MET, GAS6, and AXL levels were significantly increased during treatment (all *p* < 0.0001). Changes in potential biomarker levels in responders and nonresponders at W5D1 and W9D1 from the baseline are shown in Table 5.

**Table 4:** Changes in potential biomarker levels over time

Potential biomarker	Baseline median concentration^a^ (pg/mL)	Fold change from baseline to W5D1^a^			Fold change from baseline to W9D1^b^		
		Median	Mean	*p* value	Median	Mean	*p* value
HGF	1133	0.98	1.04	0.26	1.01	1.02	0.60
MET	17.55^c^	1.12	1.14	< 0.001	1.14	1.17	< 0.001
GAS6	23,063	1.48	1.47	< 0.001	1.29	1.32	< 0.001
AXL	33,859	1.29	1.29	< 0.001	1.28	1.35	< 0.001

*AXL* AXL receptor tyrosine kinase, *GAS6* growth arrest-specific 6, *HGF* hepatocyte growth factor, *MET* MET receptor tyrosine kinase, *W5D1* week 5 day 1, *W9D1* week 9 day 1

^a^*N* = 34; one patient who missed baseline biomarker measurements was omitted from the analysis

^b^*N* = 33; two patients who missed biomarker measurements at baseline or W9D1 were omitted from the analysis

^c^The baseline median concentration of MET was measured in ng/mL

**Table 5:** Changes in potential biomarker levels in responders and nonresponders

Potential biomarker	Group by response	Fold change from baseline to			
		W5D1		W9D1	
		Mean	*p* value^a^	Mean	*p* value^a^
HGF	Responder	1.08	0.24	1.06	0.47
	Nonresponder	0.96		0.98	
MET	Responder	1.15	0.14	1.19	0.19
	Nonresponder	1.05		1.10	
GAS6	Responder	1.48	0.82	1.31	0.48
	Nonresponder	1.51		1.40	
AXL	Responder	1.29	0.64	1.35	0.89
	Nonresponder	1.33		1.37	

*AXL* AXL receptor tyrosine kinase, *GAS6* growth arrest-specific 6, *HGF* hepatocyte growth factor, *MET* MET receptor tyrosine kinase, *W5D1* week 5 day 1, *W9D1* week 9 day 1

^a^*p* values represent comparison of fold change between responder (*n* = 25) versus nonresponder (*n* = 6); one responder patient who missed baseline biomarker measurements was omitted from the analysis

**Fig. 4:**
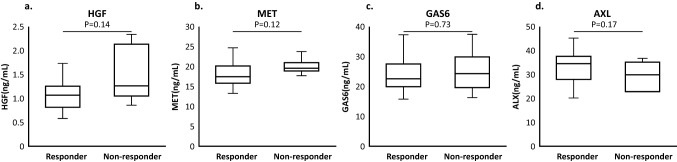
Box plots of potential biomarker levels (*n* = 31 [responders, *n* = 25; nonresponders, *n* = 6]) on W1D1 i.e. baseline. Responders: CR + PR + SD with tumor shrinkage, Nonresponders: SD with tumor enlargement + PD. **a** HGF; **b** MET; **c** GAS6; **d** AXL. Error bars indicate ± standard deviation (excluding outliers). *AXL* AXL receptor tyrosine kinase, *CR* complete response, *GAS6* growth arrest-specific 6, *HGF* hepatocyte growth factor, *MET* MET receptor tyrosine kinase, *PD* progressive disease, *PR* partial response, *SD* stable disease

As shown in Fig. 4, 31/33 patients were categorized into two groups by the BOR and the best change in the target lesion sum of diameter (SoD) (Supplementary Fig. 2). Baseline levels of potential biomarkers were shown for each patient group, the responder group (*n* = 25, CR + PR + SD with tumor shrinkage) or the nonresponder group (*n* = 6, SD with tumor enlargement + PD). Baseline levels of HGF, MET, and GAS6 tended to be lower in the responder group than the nonresponder group; however, these trends did not reach significance. Conversely, AXL levels tended to be lower in the nonresponders than the responders with no significant difference.

Patient distributions from each response group are shown in Table 6. In the nonresponder group, many patients showed higher HGF levels than the median (1114 pg/mL) at baseline. Four out of 5 (80%) nonresponders showed higher HGF levels than 1150 pg/mL, which has been reported to correlate with high-grade tumors and poor survival [17]. MET levels showed similar tendency; all nonresponders indicated higher MET levels than the median (17.64 ng/mL) at baseline.

**Table 6:** Treatment response by baseline biomarker levels dichotomized at the median

	HGF, *n* (%)		MET, *n* (%)		GAS6, *n* (%)		AXL, *n* (%)	
Median at baseline (pg/mL)	1114		17.64^c^		22,857		33,194	

	Low^b^	High^b^	Low^b^	High^b^	Low^b^	High^b^	Low^b^	High^b^
All patients, *n* = 33^a^	16 (48.5)	17 (51.5)	16 (48.5)	17 (51.5)	16 (48.5)	17 (51.5)	16 (48.5)	17 (51.5)
Responders, *n* = 25	15 (60.0)	10 (40.0)	14 (56.0)	11 (44.0)	13 (52.0)	12 (48.0)	11 (44.0)	14 (56.0)
Nonresponders, *n* = 6	1 (16.7)	5 (83.3)	0 (0.0)	6 (100.0)	3 (50.0)	3 (50.0)	4 (66.7)	2 (33.3)

*AXL* AXL receptor tyrosine kinase, *GAS6* growth arrest-specific 6, *HGF* hepatocyte growth factor, *MET* MET receptor tyrosine kinase

^a^Two patients who missed radiographic tumor assessment or biomarker measurements were omitted from the analysis

^b^Low < median; high ≥ median

^c^The baseline median concentration of MET was measured in ng/mL

## Discussion

This study was conducted to bridge the results of the cabozantinib arm in the METEOR trial to Japanese patients with a reasonable sample size set for statistical evaluation of IRC-assessed ORR. Support for the long-term efficacy and safety findings for cabozantinib in Japanese patients with ccRCC who had received at least one prior VEGFR-TKI was shown. The IRC-assessed ORR (25.7% [90% CI 14.1–40.6]) and median PFS (11.1 months [95% CI 7.4–18.4]) were numerically higher than those of the cabozantinib arm (*n* = 330) in the METEOR trial, whereby ORR was 17% (95% CI 13–22) and median PFS (primary endpoint) was 7.4 months (95% CI 6.6–9.1) [14]. TEAEs observed in more than or equal to 10% of patients were evaluated in the METOER trial and this study (Table 2). Diarrhea, hypertension and palmar-plantar erythrodysesthesia syndrome were observed as the major adverse events under cabozantinib treatment in both studies. The other safety profiles were also similar between the studies. Overall, safety was considered to be manageable [14].

Almost half of the enrolled patients (15/35, 42.9%) had received IO agents with or without a TKI before starting the study protocol. IO agents have been evolving the treatment of cancer worldwide since the approval of the ipilimumab in 2011 [18]. IO agents are now recommended as the first-line treatment option for patients with RCC in many countries, including Japan [19, 20]. There is an exploratory result from the pooled analysis of the cabozantinib arm of the METEOR trial and this Japanese phase 2 study, in which ORR was 21.2% (95% CI 9.0–38.9%) and 17.2% (95% CI 13.3–21.7%) in patients with prior-IO and no prior-IO, respectively [21]. Interest in using subsequent therapies after IO agents and combination therapies using IO agents has been raised. In this study, no stratified analysis with treatment sequences has been conducted, therefore, no additional information was obtained.

Predictive biomarkers associated with cabozantinib treatment responses will support the identification of patients who may benefit from treatment. Currently, no plasma biomarkers that are consistently predictive or prognostic for an improved benefit with cabozantinib treatment have been found. For example, on-treatment changes in HGF appeared prognostic for improved PFS or OS with cabozantinib in univariate analyses, but not independently prognostic in multivariate analyses in the METEOR trial [15].

In our study described here, an exploratory examination of four potential biomarkers suggested in the METEOR trial was undertaken using ELISA and plasma samples, which is a feasible method in clinical practice. Plasma levels of MET and AXL, which are membrane receptors, were assessed as surrogate markers for these receptors. Fold changes from baseline in the median/mean at week 5 were similar to the results in the cabozantinib arm of the METEOR trial [15]. In addition, moderate elevations of MET, GAS6, and AXL were observed at week 5 and were still higher than the baseline levels at week 9 (Table 4). The clinical meaning of these elevations remains unclear. HGF levels were stable during this period. We assessed the on-treatment changes of these factors for responders and nonresponders; however, minimal difference was observed between groups (Table 5). As an additional consideration, we used different definition for responders and nonresponders (Supplementary Fig. 3, Supplementary Table 2 and 3). Responders were composed of CR and PR, PD and SD were included in nonresponders. With this categorization, HGF was significantly lower in responders. HGF might be a predictive biomarker of cabozantinib treatment. All the other factors showed similar trends with those when SD with tumor shrinkage was included in responders.

Tanimoto et al., previously reported that serum HGF levels were significantly higher in patients with ccRCC (1070.7 pg/mL, *n* = 45) than healthy patients (728 pg/mL, *n* = 45), and HGF > 1150 pg/mL correlated with poor survival [17]. In the METEOR trial, PFS and OS were longer with cabozantinib versus everolimus in both patient populations with low or high baseline HGF, suggesting a benefit with cabozantinib treatment irrespective of baseline HGF levels [15]. In this study, we evaluated tumor shrinkage as the indicator of cabozantinib treatment response and the correlation of baseline HGF levels (Fig. 4). Responders tended to show lower baseline HGF levels, which was similar to the results from METEOR.

In summary, support for the long-term efficacy and safety findings for cabozantinib in Japanese patients with ccRCC who had received at least one prior VEGFR-TKI was shown in our study. Responders tended to show lower baseline HGF levels.

## Supplementary Information

Below is the link to the electronic supplementary material.

Supplementary file1 (DOCX 138 KB)

## Data Availability

The data sets, including the redacted study protocol, redacted statistical analysis plan, and individual participant data supporting the results reported in this article, will be made available within 3 months from initial request, to researchers who provide a methodologically sound proposal. The data will be provided after de-identification, in compliance with applicable privacy laws, data protection, and requirements for consent and anonymization.

## References

[1] Inamura K (2017). Renal cell tumors: understanding their molecular pathological epidemiology and the 2016 WHO classification. Int J Mol Sci.

[2] Padala SA, Barsouk A, Thandra KC (2020). Epidemiology of renal cell carcinoma. World J Oncol.

[3] Barry RE, Krek W (2004). The von Hippel-Lindau tumour suppressor: a multi-faceted inhibitor of tumourigenesis. Trends Mol Med.

[4] Jonasch E, Walker CL, Rathmell WK (2021). Clear cell renal cell carcinoma ontogeny and mechanisms of lethality. Nat Rev Nephrol.

[5] Kaelin WG (2008). The von Hippel-Lindau tumour suppressor protein: O2 sensing and cancer. Nat Rev Cancer.

[6] Rini BI, Atkins MB (2009). Resistance to targeted therapy in renal-cell carcinoma. Lancet Oncol.

[7] Rankin EB, Fuh KC, Castellini L (2014). Direct regulation of GAS6/AXL signaling by HIF promotes renal metastasis through SRC and MET. Proc Natl Acad Sci USA.

[8] Choueiri TK, Motzer RJ (2017). Systemic therapy for metastatic renal-cell carcinoma. N Engl J Med.

[9] Na X, Wu G, Ryan CK (2003). Overproduction of vascular endothelial growth factor related to von Hippel-Lindau tumor suppressor gene mutations and hypoxia-inducible factor-1 alpha expression in renal cell carcinomas. J Urol.

[10] Motzer R, Alekseev B, Rha SY (2021). Lenvatinib plus pembrolizumab or everolimus for advanced renal cell carcinoma. N Engl J Med.

[11] Bergers G, Hanahan D (2008). Modes of resistance to anti-angiogenic therapy. Nat Rev Cancer.

[12] Qu L, Ding J, Chen C (2016). Exosome-transmitted lncARSR promotes sunitinib resistance in renal cancer by acting as a competing endogenous RNA. Cancer Cell.

[13] Yakes FM, Chen J, Tan J (2011). Cabozantinib (XL184), a novel MET and VEGFR2 inhibitor, simultaneously suppresses metastasis, angiogenesis, and tumor growth. Mol Cancer Ther.

[14] Choueiri TK, Escudier B, Powles T (2016). Cabozantinib versus everolimus in advanced renal cell carcinoma (METEOR): final results from a randomised, open-label, phase 3 trial. Lancet Oncol.

[15] Powles T, Choueiri TK, Motzer RJ (2021). Outcomes based on plasma biomarkers in METEOR, a randomized phase 3 trial of cabozantinib vs everolimus in advanced renal cell carcinoma. BMC Cancer.

[16] Tomita Y, Tatsugami K, Nakaigawa N (2020). Cabozantinib in advanced renal cell carcinoma: A phase II, open-label, single-arm study of Japanese patients. Int J Urol.

[17] Tanimoto S, Fukumori T, El-Moula G (2008). Prognostic significance of serum hepatocyte growth factor in clear cell renal cell carcinoma: comparison with serum vascular endothelial growth factor. J Med Invest.

[18] Hodi FS, O’Day SJ, McDermott DF (2010). Improved survival with ipilimumab in patients with metastatic melanoma. N Engl J Med.

[19] Japanese guideline: Clinical Practice Guideline for Renal Cancer 2017 (2019 updated). Available from URL: https://www.urol.or.jp/lib/files/other/guideline/33_renal_cancer_2017_rev2020_info.pdf. Accessed Jun 2020

[20] National Comprehensive Cancer Network . NCCN Clinical Practice Guidelines in Oncology (NCCN Guidelines): Kidney Cancer version 1.2023. Available from URL: https://www.nccn.org/professionals/physician_gls/pdf/kidney.pdf. Accessed 17 Jun 2022

[21] Oya M, Tamada S, Tatsugami K et al (2020) ASCO Annual Meeting Abstract 5089

