# Supplementary material for: Final efficacy and safety results and biomarker analysis of a phase 2 study of cabozantinib in Japanese patients with advanced renal cell carcinoma
Source: Int J Clin Oncol. 2023 Jan 3;28(3):416–26. doi: 10.1007/s10147-022-02283-w (PMC9988754; doi:10.1007/s10147-022-02283-w)
Supplement: Supplementary file 1 — Supplementary file1 (DOCX 138 KB) [file 10147_2022_2283_MOESM1_ESM.docx]

**Supplementary information**





**Supplementary Fig. 1** Mean change from baseline in total NCCN-FKSI-19 scores (*n = 35).* Error bars indicate ± SD)

*FKSI-19* Functional Assessment of Cancer Therapy-Kidney Cancer Symptom Index, *NCCN* National Comprehensive Cancer Network, *SD* standard deviation

**
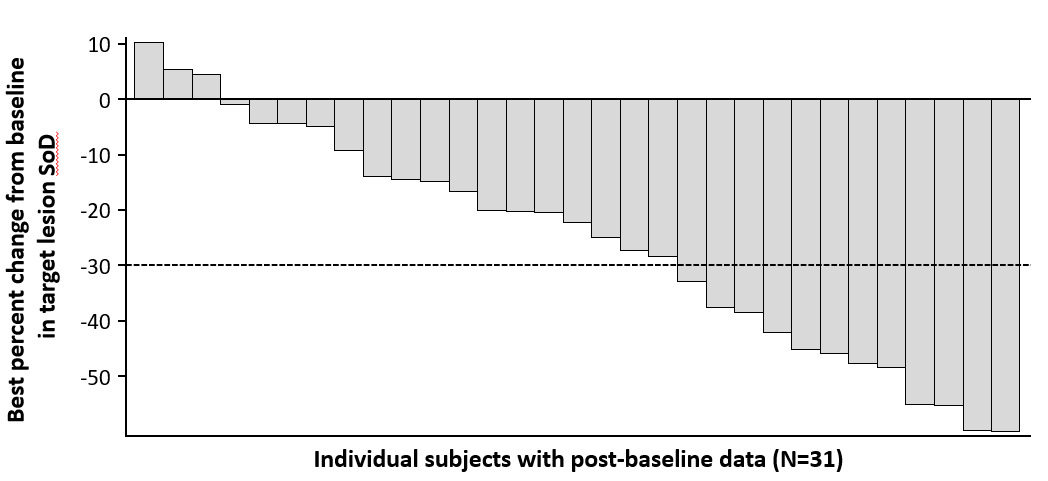
**

**Supplementary Fig. 2** Waterfall plot of best percentage change in target lesion size by IRC.

One patient with no post-baseline analysis and three patients without target lesions by IRC were excluded from the analysis.

*IRC* independent review committee, *SoD* sum of diameters

**
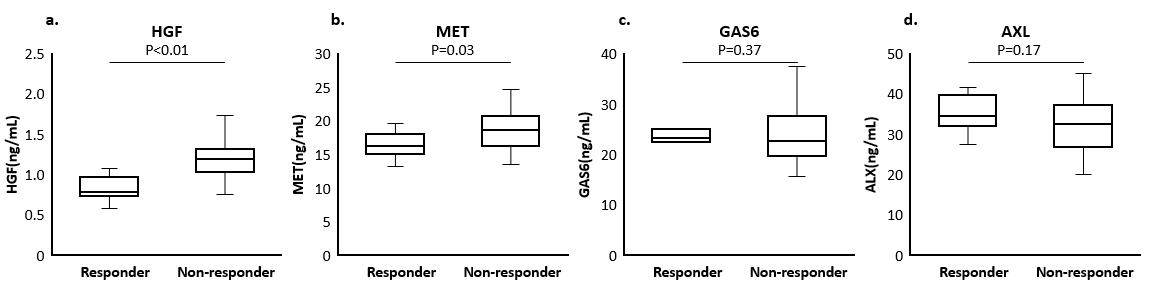

Supplementary Fig. 3** Box plots of potential biomarker levels (n = 31 [responders, n = 7; nonresponders, n = 24]) on W1D1 i.e. baseline. Responders: CR + PR, Nonresponders: SD + PD. **a** HGF; **b** MET; **c** GAS6; **d** AXL. Error bars indicate ± standard deviation (excluding outliers) .

*AXL* AXL receptor tyrosine kinase, *CR* complete response, *GAS6* growth arrest-specific 6, *HGF* hepatocyte growth factor, *MET* MET receptor tyrosine kinase, *PD* progressive disease, *PR* partial response, *SD* stable disease

**Supplementary Table 1** Mean changes from baseline in FKSI-19 scores by visit

| **Variable** | ***N*** | **Mean change (SD)** | **95% CI** |
| --- | --- | --- | --- |
| **NCCN-FKSI-19 Total Score** | | | |
| Week 5 | 35 | −5.9 (10.40) | −9.49, −2.34 |
| Week 9 | 34 | −4.4 (9.94) | −7.85, −0.91 |
| Week 13 | 31 | −2.2 (10.58) | −6.11, 1.65 |
| Week 17 | 28 | −2.0 (8.67) | −5.40, 1.33 |
| Week 21 | 28 | −1.2 (7.96) | −4.30, 1.87 |
| Week 25 | 27 | −0.3 (9.37) | −3.96, 3.45 |
| Week 33 | 25 | −1.2 (10.23) | −5.38, 3.06 |
| Week 41 | 23 | −2.2 (10.38) | −6.71, 2.27 |
| Week 49 | 22 | −1.0 (7.78) | −4.49, 2.40 |
| Week 57 | 19 | −1.9 (10.65) | −7.03, 3.24 |
| Week 65 | 15 | −5.3 (10.43) | −11.04, 0.51 |
| Week 73 | 15 | −4.1 (12.33) | −10.96, 2.70 |
| Week 81 | 14 | −4.6 (10.17) | −10.44, 1.30 |
| Week 89 | 13 | −3.8 (11.00) | −10.42, 2.88 |
| Week 97 | 11 | −4.7 (10.85) | −12.02, 2.56 |
| Week 105 | 5 | −3.6 (11.72) | −18.15, 10.95 |
| Week 113 | 1 | −7.0 | − |
| **Disease-related symptoms**  **Physical score** | | | |
| Week 5 | 35 | −3.4 (5.87) | −5.42, −1.38 |
| Week 9 | 34 | −3.0 (6.08) | −5.12, −0.88 |
| Week 13 | 31 | −2.0 (5.34) | −3.93, −0.01 |
| Week 17 | 28 | −1.4 (4.47) | −3.09, 0.37 |
| Week 21 | 28 | −0.9 (4.77) | −2.78, 0.92 |
| Week 25 | 27 | −0.2 (5.19) | −2.28, 1.83 |
| Week 33 | 25 | −0.6 (6.01) | −3.12, 1.84 |
| Week 41 | 23 | −1.7 (7.00) | −4.68, 1.38 |
| Week 49 | 22 | −0.5 (5.45) | −2.87, 1.96 |
| Week 57 | 19 | −0.3 (6.40) | −3.40, 2.77 |
| Week 65 | 15 | −3.6 (5.26) | −6.51, −0.69 |
| Week 73 | 15 | −2.2 (6.54) | −5.82, 1.42 |
| Week 81 | 14 | −2.9 (6.34) | −6.59, 0.73 |
| Week 89 | 13 | −2.2 (6.78) | −6.25, 1.94 |
| Week 97 | 11 | −1.4 (6.79) | −5.92, 3.20 |
| Week 105 | 5 | −2.8 (7.26) | −11.81, 6.21 |
| Week 113 | 1 | −7.0 | − |
| **Emotional score** | | | |
| Week 5 | 35 | 0.5 (1.09) | 0.14, 0.89 |
| Week 9 | 34 | 0.3 (1.19) | −0.15, 0.68 |
| Week 13 | 31 | 0.6 (1.06) | 0.19, 0.97 |
| Week 17 | 28 | 0.4 (1.10) | 0.00, 0.86 |
| Week 21 | 28 | 0.3 (1.08) | −0.17, 0.67 |
| Week 25 | 27 | 0.4 (1.39) | −0.18, 0.92 |
| Week 33 | 25 | 0.3 (1.34) | −0.27, 0.83 |
| Week 41 | 23 | 0.3 (1.29) | −0.30, 0.82 |
| Week 49 | 22 | 0.0 (1.29) | −0.62, 0.53 |
| Week 57 | 19 | −0.6 (1.46) | −1.34, 0.07 |
| Week 65 | 15 | −0.4 (1.45) | −1.21, 0.41 |
| Week 73 | 15 | −0.1 (1.36) | −0.88, 0.62 |
| Week 81 | 14 | −0.1 (1.00) | −0.65, 0.50 |
| Week 89 | 13 | −0.1 (1.04) | −0.70, 0.55 |
| Week 97 | 11 | −0.1 (0.83) | −0.65, 0.47 |
| Week 105 | 5 | −1.0 (1.22) | −2.52, 0.52 |
| Week 113 | 1 | 0.0 | – |
| **Treatment side effects score** | | | |
| Week 5 | 35 | −1.9 (2.53) | −2.81, −1.07 |
| Week 9 | 34 | −2.1 (2.19) | −2.91, −1.38 |
| Week 13 | 31 | −1.7 (2.24) | −2.53, −0.89 |
| Week 17 | 28 | −2.5 (2.46) | −3.42, −1.51 |
| Week 21 | 28 | −2.0 (2.15) | −2.80, −1.13 |
| Week 25 | 27 | −1.6 (2.02) | −2.39, −0.79 |
| Week 33 | 25 | −1.6 (2.24) | −2.52, −0.68 |
| Week 41 | 23 | −2.1 (2.64) | −3.23, −0.94 |
| Week 49 | 22 | −2.4 (2.91) | −3.70, −1.12 |
| Week 57 | 19 | −2.5 (2.41) | −3.64, −1.31 |
| Week 65 | 15 | −2.5 (2.70) | −3.96, −0.97 |
| Week 73 | 15 | −2.6 (3.09) | −4.31, −0.89 |
| Week 81 | 14 | −2.0 (2.54) | −3.47, −0.53 |
| Week 89 | 13 | −1.7 (2.81) | −3.39, 0.01 |
| Week 97 | 11 | −2.6 (2.73) | −4.47, −0.80 |
| Week 105 | 5 | −1.6 (3.13) | −5.49, 2.29 |
| Week 113 | 1 | 2.0 | – |
| **Function/well-being score** | | | |
| Week 5 | 35 | −1.1 (5.12) | −2.84, 0.67 |
| Week 9 | 34 | 0.5 (4.59) | −1.10, 2.10 |
| Week 13 | 31 | 0.9 (5.35) | −1.09, 2.83 |
| Week 17 | 28 | 1.4 (4.79) | −0.50, 3.21 |
| Week 21 | 28 | 1.4 (5.01) | −0.52, 3.37 |
| Week 25 | 27 | 1.2 (4.72) | −0.68, 3.05 |
| Week 33 | 25 | 0.8 (4.72) | −1.15, 2.75 |
| Week 41 | 23 | 1.3 (4.48) | −0.68, 3.20 |
| Week 49 | 22 | 1.9 (4.20) | 0.00, 3.73 |
| Week 57 | 19 | 1.5 (4.99) | −0.88, 3.93 |
| Week 65 | 15 | 1.2 (5.88) | −2.06, 4.46 |
| Week 73 | 15 | 0.8 (6.16) | −2.61,4.21 |
| Week 81 | 14 | 0.4 (5.98) | −3.03, 3.88 |
| Week 89 | 13 | 0.2 (5.13) | −2.95, 3.25 |
| Week 97 | 11 | −0.6 (5.03) | −4.01, 2.74 |
| Week 105 | 5 | 1.8 (5.63) | −5.19, 8.79 |
| Week 113 | 1 | −2.0 | − |

*FKSI-19* Functional Assessment of Cancer Therapy-Kidney Cancer Symptom Index 19 Item Version, *SD* standard deviation

**Supplementary Table 2** Changes in potential biomarker levels in responders and nonresponders

| Potential biomarker | Group by response | Fold change from baseline to | | | |
| --- | --- | --- | --- | --- | --- |
|  |  | W5D1 | | W9D1 | |
|  |  | Mean | *p* value^a^ | Mean | *p* value^a^ |
| HGF | Responder | 1.21 | 0.02 | 1.18 | <0.01 |
|  | Nonresponder | 1.01 |  | 1.00 |  |
| MET | Responder | 1.16 | 0.55 | 1.17 | 0.95 |
|  | Nonresponder | 1.12 |  | 1.17 |  |
| GAS6 | Responder | 1.41 | 0.37 | 1.38 | 0.57 |
|  | Nonresponder | 1.51 |  | 1.32 |  |
| AXL | Responder | 1.24 | 0.37 | 1.39 | 0.69 |
|  | Nonresponder | 1.31 |  | 1.34 |  |

*AXL* AXL receptor tyrosine kinase, *GAS6* growth arrest-specific 6, *HGF* hepatocyte growth factor, *MET* MET receptor tyrosine kinase, *W5D1* week 5 day 1, *W9D1* week 9 day 1

^a^*p* values represent comparison of fold change between responder (CR+PR, *n* = 7) versus nonresponder (SD+PD, *n* = 24); one responder patient who missed baseline biomarker measurements was omitted from the analysis

**Supplementary Table 3** Treatment response by baseline biomarker levels dichotomized at the median

|  | HGF, *n* (%) | | MET, *n* (%) | | GAS6, *n* (%) | | | | AXL, *n* (%) | |
| --- | --- | --- | --- | --- | --- | --- | --- | --- | --- | --- |
| Median at baseline (pg/mL) | 1114 | | 17.64^c^ | | | 22857 | | | 33194 | |
|  | Low^b^ | High^b^ | Low^b^ | High^b^ | | Low^b^ | High^b^ | Low^b^ | | High^b^ |
| All patients, *n* = 33^a^ | 16  (48.5) | 17  (51.5) | 16  (48.5) | 17  (51.5) | | 16  (48.5) | 17  (51.5) | 16  (48.5) | | 17 (51.5) |
| Responders, *n* = 7 | 7  (100.0) | 0  (0.0) | 5  (71.4) | 2  (28.6) | | 3  (42.9) | 4  (57.1) | 4  (57.1) | | 3 (42.9) |
| Nonresponders, *n* = 24 | 9  (37.5) | 15  (62.5) | 9  (37.5) | 15  (62.5) | | 13  (54.2) | 11  (45.8) | 11  (45.8) | | 13  (54.2) |

*AXL* AXL receptor tyrosine kinase, *GAS6* growth arrest-specific 6, *HGF* hepatocyte growth factor, *MET* MET receptor tyrosine kinase

^a^Two patients who missed radiographic tumor assessment or biomarker measurements were omitted from the analysis

^b^Low < median; high ≥ median

^c^The baseline median concentration of MET was measured in ng/mL

Responders: CR+PR, Nonresponders : SD+PD
